# Epidemiology of postnatal depression and its associated factors in Africa: A systematic review and meta-analysis

**DOI:** 10.1371/journal.pone.0231940

**Published:** 2020-04-28

**Authors:** Abel Fekadu Dadi, Temesgen Yihunie Akalu, Adhanom Gebreegziabher Baraki, Haileab Fekadu Wolde

**Affiliations:** 1 Department of Epidemiology and Biostatistics, Institute of Public Health, College of Medicine & Health Sciences, University of Gondar, Gondar, Ethiopia; 2 College of Medicine and Public Health, Discipline of Public Health, Flinders University, Adelaide, Australia; Monash University, AUSTRALIA

## Abstract

**Introduction:**

Postnatal depression (PND) is a major cause of negative health-related behaviors and outcomes during infancy, childhood and adolescent period. In Africa, the burden of postnatal depression is high. However, it is under-investigated hence under-treated. To fill this information gap and to advise further interventions, we aimed at analyzing its epidemiology in Africa.

**Methods:**

We searched observational studies conducted in Africa and published in between 01/01/2007 and 30/06/2018 in CINHAL, MEDLINE, PsycINFO, Psychiatry online, PubMed, SCOPES, and Emcare databases. We assessed the quality of the studies using the Newcastle Ottawa Scale (NOS) and included studies with good quality. We evaluated the heterogeneity using the Higgins *I*^2^ statistics. We used a random-effects model to pool estimates. We assessed publication bias using the funnel plot and Egger's test statistics and adjusted using Tweedie’s and Duval Trim and Fill analysis. The protocol has been registered in the PROSPERO (Protocol No. CRD42018100461).

**Results:**

Nineteen studies involving 40,953 postnatal mothers were part of this systematic review and meta-analysis. The overall pooled prevalence of PND was 16.84% (95% CI: 14.49% –19.19%). The odds of having PND was higher among women with a poor obstetric condition (POR = 2.11; 95% CI: 1.11–4.01) and history of adverse birth and infant health outcomes (POR = 2.85; 95% CI: 1.29–6.25). Having a history of common mental health disorders (POR = 2.47; 95% CI: 1.51–4.04), poor social support (POR = 2.06; 95% CI: 1.05–4.05), lower economic status (POR = 2.38; 95% CI: 1.75–3.23), and those who had exposure to a different form of intimate partner violence (POR = 2.87; 95% CI: 1.60–5.16) had higher odds of PND.

**Conclusion:**

While robust prevalence studies are scarce, our review indicated a high prevalence rate of postnatal depression. The analysis also identified postpartum women at increased risk of PND. Therefore, there is a need to design and escalate comprehensive strategies to decrease its burden, focusing on those women at risk of PND.

## Background

Postnatal depression (PND) is a major depressive episode that occurs after 15 days following delivery and continues for one year [1, 2]. The burden of PND is a significant public health concern in low and middle-income countries [3]. According to the World Health Organization (WHO) 2017 report, more than 322 million people had depression, and of this, about 29.9 million (9%) were in Africa [4]. Globally, the prevalence of PND increased by 18.4% in the year from 2005 to 2015[5]. A PND prevalence of 19.8% was reported in a systematic review conducted in 17 low-and middle-income countries [6]. Africa constitutes a higher burden of PND while on average, one in every ten women had depression [7].

Postnatal depression increases maternal morbidity [8, 9], social problems [10], physical damage [11], and leads to suicide [12]. Similarly, there is a strong relationship between maternal wellbeing and child development [13]. Untreated PND leads to infant growth retardation [14, 15], poor child survival [16], impaired child development [17, 18], behavioral changes [19], repeated diarrheal disease [20], and under-nutrition [21, 22]. Likewise, PND can also affect postnatal mothers' quality of life [23]. In the low-and middle-income countries, about 80% of patients with psychological, neurological, and substance use conditions do not access services [24]. The World Health Organization (WHO) has launched the Mental Health Gap Action Program (mhGAP) that aimed at integrating mental health interventions with the existing maternal health services [7, 24].

The prevalence of PND can be affected by factors such as age [25], low household income [26], unwanted pregnancy [27, 28], having emesis during pregnancy [29], depression during the first two trimesters of pregnancy [27, 30, 31], psychiatric problems during pregnancy [32], delivery by cesarean section [33], lack of satisfaction in marital status [28, 31], co-morbid condition like anemia [34], gestational Diabetic Mellitus (DM) [27], social factors [25, 31], low self-esteem [35], prematurity [23], and behavioral factors such as smoking [36].

Despite its burden, depression during the postnatal period is still under-investigated and undertreated in Africa [37]. Reviews that have been conducted in low-and middle-income countries did not include representative studies from Africa, and they were also outdated. There is a systematic review conducted on perinatal psychological disorders in Africa that incorporated postnatal depression as one specific objective [38]. Therefore, we did the current systematic review and meta-analysis to present the pooled estimate and risk factors of PND in Africa precisely.

## Methods

### Data source and searches

We systematically searched for articles conducted in CINHAL, MEDLINE, PsycINFO, psychiatry online, PubMed, SCOPES, and Emcare databases. The following search terms combined with Boolean operators, wildcard and truncation were used: “postpartum women”, “lactating women”, “postnatal mothers”, “major depression”, “psychological morbidity”, “depressive symptoms”, “major depressive disorder”, “depressed mood”, “clinical depression”, and “depression”. Example of the search strategy in MEDLINE:

(exp POSTPARTUM DEPRESSION/) or (Depress*.tw,id.) AND (postnat* or postnatal wom?n or postpartum wom?n).tw,id.) [mp = title, abstract, original title, name of substance word, subject heading word, keyword heading word, protocol supplementary concept word, rare disease supplementary concept word, unique identifier, synonyms]) AND ((exp Psychosocial Factors/ or exp Risk Factors/) or (risk*.tw,id.)) AND ((prospective cohort* or retrospective cohort* follow up* or longitudinal* or cross-sectional* or case-control* or nested-case control).mp.): all Sort by: PublicationDateFilters: Publication date from 2007/01/01 to 2017/12/31; Humans; English; Female; Field: Title/Abstract

#### Included studies

We included all observational studies conducted in Africa, written in the English language, conducted from January 1, 2007, to May 30, 2018, and investigated postnatal depression and/or its associated factors. Studies were included in the current review if they used a standardized and validated tool to diagnose depression and diagnosed depression in the first two years of childbirth.

#### Excluded studies

This review excluded studies with poor quality and those conducted in a high-risk population (HIV).

### Study selection

The title of all retrieved articles was screened, and those fit were imported to endnote software. The primary author did literature searching, title review, and duplicate removal. After excluding duplicated articles, abstracts and their full-text were independently reviewed by two groups of authors: group one (TYA, AFD) and group two (AGB, HFW). Differences were resolved through discussion. Articles deemed relevant during the abstract and full- text review were assessed for quality assessment.

### Data extraction and quality assessment

The quality of included studies was assessed using the Newcastle Ottawa Scale (NOS) by two independent reviewers (TYA and AFD), and the third reviewer (HFW) was a tie break in case of discordance between the two reviewers. Articles that scored ≥7 points were considered as “good” quality and were included in the final review and meta-analysis [39]. Name of author, year of publication, country, study setting, study design, sample size, time of screening, tools used for screening depression, and estimates (prevalence, associated factors with their adjusted odds ratio and 95% confidence interval) information were abstracted and presented in a table.

### Data synthesis and analysis

Data abstraction was done using a Microsoft Excel spreadsheet and was exported to Stata 14 for analysis. The prevalence of PND and odds ratios of risk factors obtained from each study were pooled after transforming the original estimates. Sub-group analysis was conducted based on the type of tool used for screening depression [40], geographical location, the income of countries, time of depression measurement, study setting, sample size, and year of publication. The magnitude of heterogeneity among included studies was quantitatively measured by *I*^*2*^ and Higgins test (p-value <0.05). Sensitivity analysis was performed for checking the presence of an influential study. Publication bias was checked using a visual inspection of the funnel plot and Egger’s regression test (P-value < 0.05). In the case of publication bias, Duval and Tweedie’s Trim and Fill analysis in the random effect model was performed as an adjustment [41]. Finally, results were presented using tables and forest plots.

### Protocol registration

The protocol for this systematic review and meta-analysis has been registered in the PROSPERO (Protocol No. CRD42018100461).

### Data reporting

**Fig 1:**
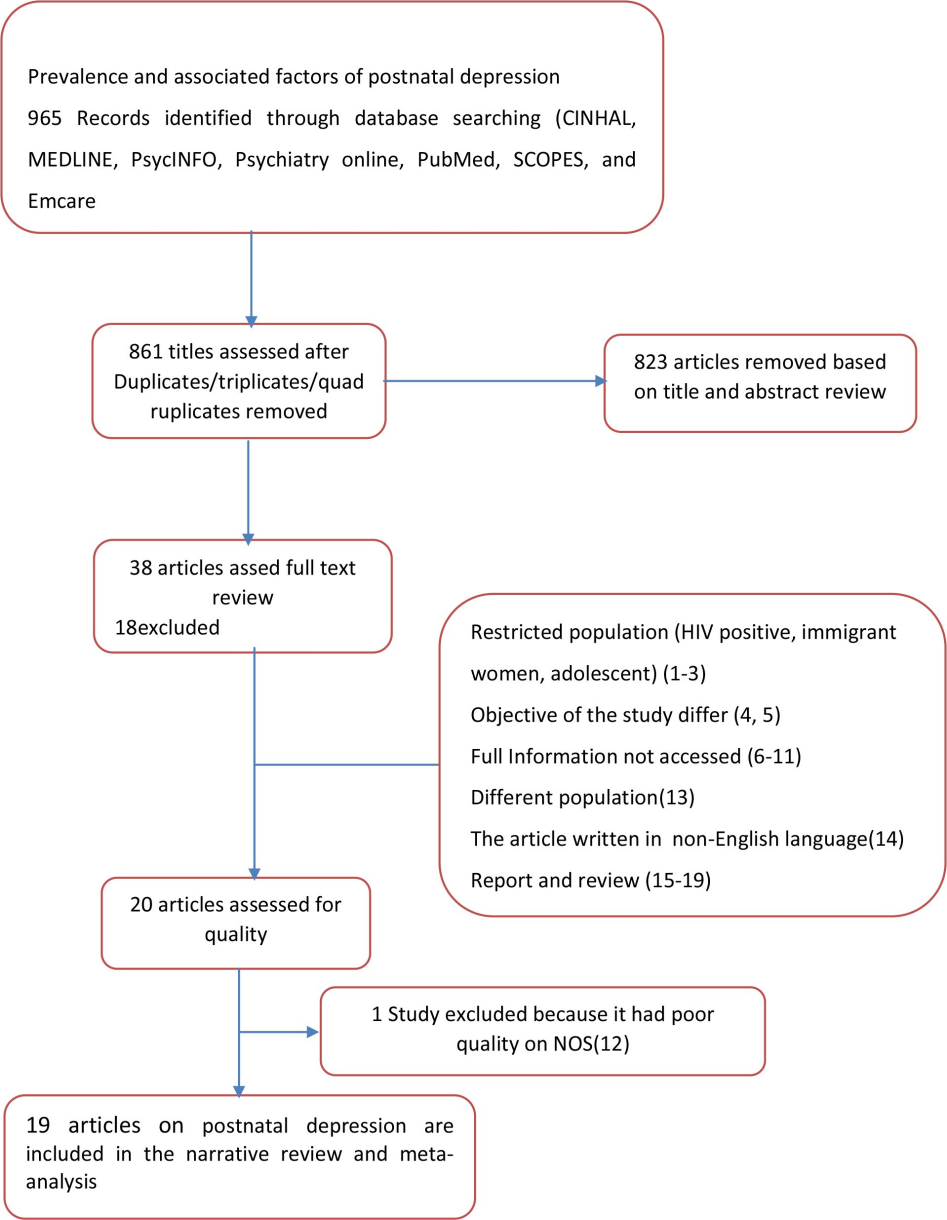
PRISMA flow diagram indicating the study selection for inclusion in the systematic review.

The PRISMA [42] statement for reporting a systematic review and meta-analysis was used to present the study inclusion, exclusion, and reasons for exclusion in a diagram (Fig 1). Finally, the reporting of this systematic review and meta-analysis result followed the Meta-analysis Of Observational Studies in Epidemiology (MOOSE) statement[43].

## Results

### Description of studies

The electronic database search retrieved 965 records and 104 records were duplicates. The titles and abstracts of 861 articles were assessed, and 823 articles were removed. Full text of 38 articles was evaluated, and 18 articles were excluded. The reason for exclusion includes: conducted on restricted population [44, 45], different objective from the current review [46, 47],lack of access to full articles[48–53], conducted in a different population with this review [54], the article was written in a non-English language [55], and were reviews and reports [56–60]. Twenty articles were assessed for quality, and one study was excluded because it had poor quality on NOS [61]. Finally, 19 articles (40,953 study participants) with good quality were included in this systematic review and meta-analysis (Fig 1).

Of 19 studies, five were from Ghana [62–66], three were from Ethiopia [7, 67], two were from Egypt [68, 69], two were from Tanzania [70, 71], two were from South Africa [72, 73], and one was from each of these countries (Malawi [74], Sudan [75], Zimbabwe [76], Zambia [77], and Cotedevior). Included studies were published in the year from 2007 to 2018 with a sample size ranging from 159 in South Africa to 16,560 in Ghana. Twelve (63.16%) and seven (36.84%) studies were cross-sectional and cohort studies, respectively. Nine studies were conducted in the community, while ten studies were conducted in health institutions. The time of screening for PND varied from 4 weeks to 56 weeks. Postpartum depression was measured using PDQ in 1 study, DSM-IV in 2 studies, EPDS in 6 studies, PHQ -9 in 7 studies, CES-D in 2 studies, and SRQ-20 in 1 study (Table 1).

**Table 1:** Characteristics of included studies: A systematic review and meta-analysis of postpartum depression in Africa.

	Author, P. year	Country	Study setting	Study design	Sample size	Screening time	Tool used	Prevalence
1.	Ramchandani PG et al. 2008	South Africa	Community	Cohort	1035	24 weeks	PDQ	16.40%
2.	Stellenberg E et al. 2016	South Africa	Community	Cross-sectional	159	6 to 14 weeks	EPDS	50.30%
3.	Stewart RC et al. 2009	Malawi	HI	Cross-sectional	501	36 weeks	DSM-IV	13.90%
4.	Hassanein I et al. 2014	Egypt	HI	Cross-sectional	290	12 weeks	EPDS	39%
5.	Mohammed ES et al. 2014	Egypt	Community	Cross-sectional	200	56 weeks	EPDS	49.50%
6.	Khalifa DS et al. 2015	Sudan	HI	Follow up	300	12 weeks	EPDS	9.20%
7.	Shamu S et al. 2016	Zimbabwe	HI	Cross-sectional	842	6 weeks	CES-D	21.40%
8.	Ndokera R et al. 2008	Zambia	Community	Cross-sectional	278	8 to 48 weeks	SRQ-20	9.70%
9.	Guo N et al 2013	Cot devoir	HI	Cohort	654	12 weeks	PHQ_9	11.80%
10.	Weobong B et al. 2017	Ghana	Community	Cohort	16,560	4 to 12 weeks	DSM-IV	3.50%
11.	Weobong B et al. 2016	Ghana	Community	Cohort	13, 360	4 weeks	PHQ-9	3.80%
12.	Guo N et al. 2013	Ghana	HI	Cohort	654	12 weeks	PHQ_9	8.90%
13.	Wemakor A et al. 2018	Ghana	Community	Cross-sectional	200	6–23 months	CES-D	33.50%
14.	Anokiye R et al. 2018	Ghana	HI	Cross-sectional	257	0–48 weeks	PHQ-9	7.00%
15.	Adamu AF et al. 2018	Ethiopia	HI	Cross sectional	618	0–6 weeks	EPDS	23.30%
16.	Azale A et al.2018	Ethiopia	Community	Cross-sectional	3147	1-12months	PHQ-9	12.23%
17.	Azale A et al. 2016	Ethiopia	Community	Cross-sectional	385	24 weeks	PHQ_9	12.13%
18.	Mahenge B et al. 2018	Tanzania	HI	Cross-sectional	500	4–36 weeks	PHQ-9	13.60%
19	Rogathi JJ et al. 2017	Tanzania	HI	Cohort	1013	40 days	EPDS	12.00%

**HI**: Health Institution, EPDS: Edinburgh Postnatal Depression scale **SRQ**: Self Reporting Questionnaire **CESD-10**: Center for Epidemiological Studies Depression Scale, **PHQ**: Patient Health Questionnaire, **DSM-V**: Diagnostic and Statistical Manual of Mental DisorderPDQ: Perceived Deficit Questionnaire.

### The pooled prevalence of postnatal depression

**Fig 2:**
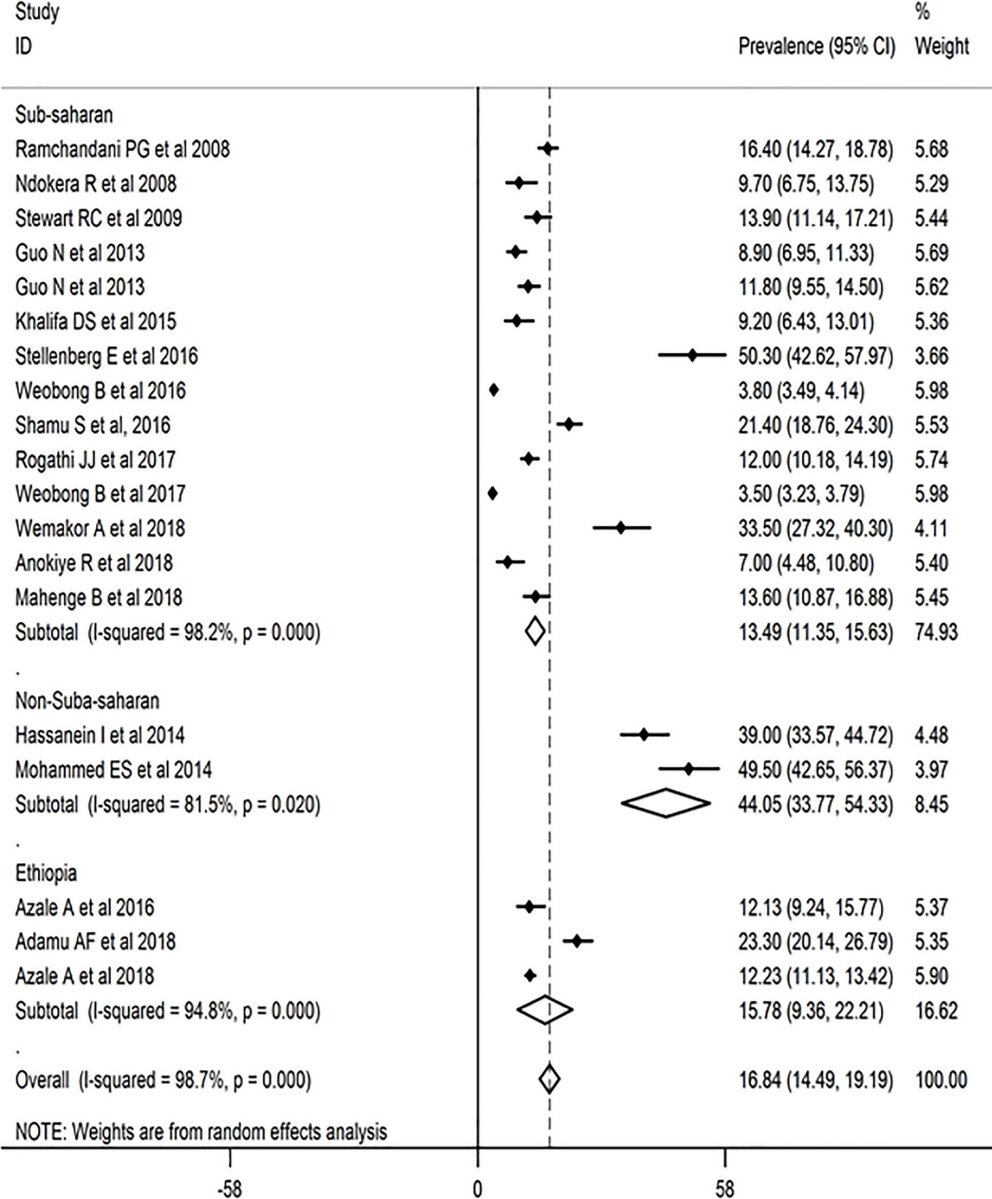
Postnatal depression prevalence in Africa, a meta-analysis, sub analyzed by geographical area (N = 19, random effect).

**Fig 3:**
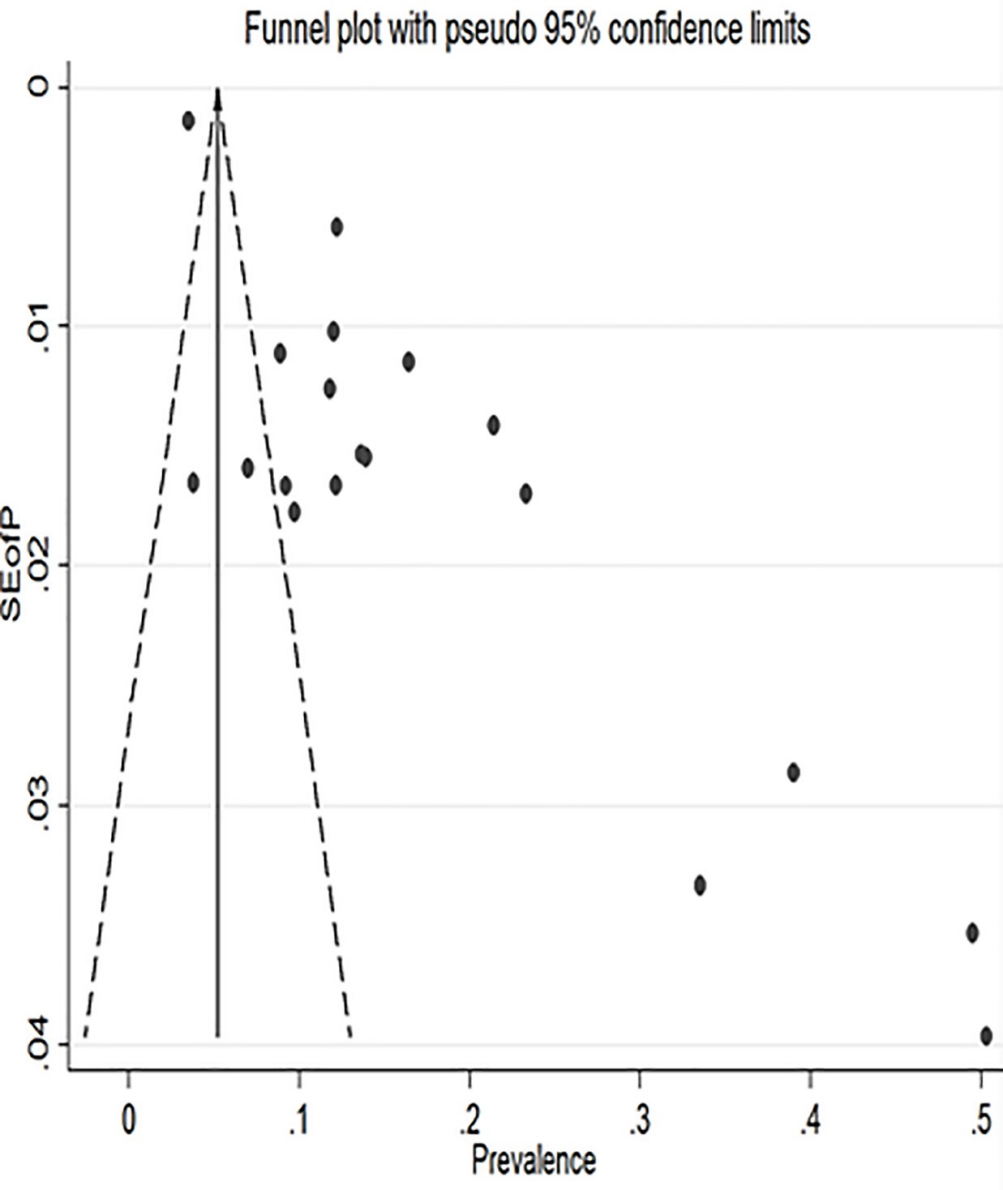
Funnel plot with pseudo 95% confidence interval limits.

**Fig 4:**
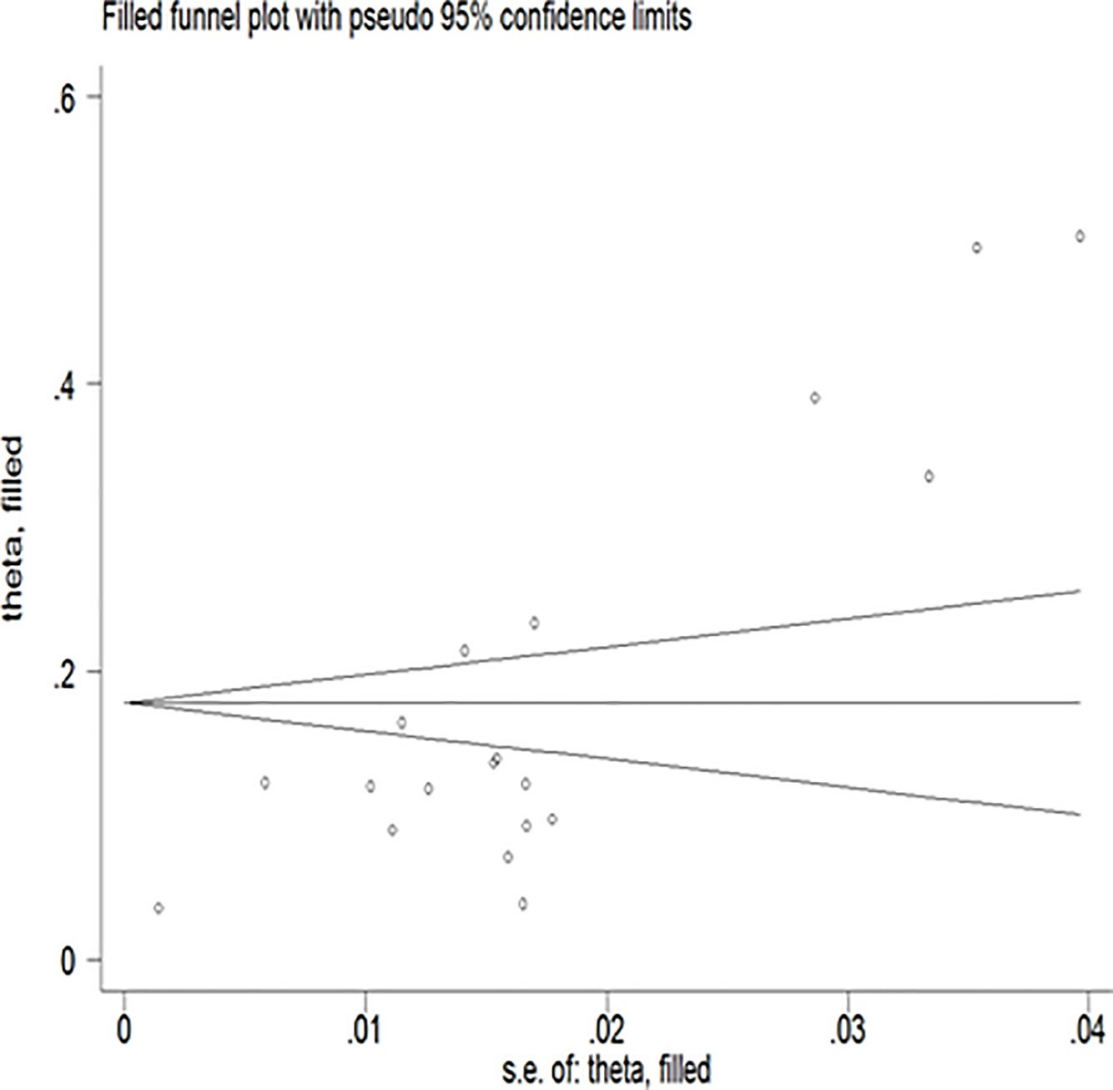
Filled funnel plot with pseudo 95% confidence interval.

The pooled prevalence of postnatal depression before adjusting for publication bias was 16.84% (95% CI: 14.49–19.19, *I*^*2*^ *=* 98.7%, Eggers test = 0.001) (Fig 2). However, after adjustment, the final pooled prevalence was found to be 17.8% (95% CI: 13.9%, 21.7%). There is no significant difference in estimates between the original and the trimmed prevalence (Figs 3 & 4). The extent of heterogeneity among the included studies was high. A meta-regression showed that the effect of the difference in sample size explained 12% (P-value = 0.09) of the total observed variation. We also further did and report estimates from a sub-analysis considering other possible sources of variations.

**Fig 5:**
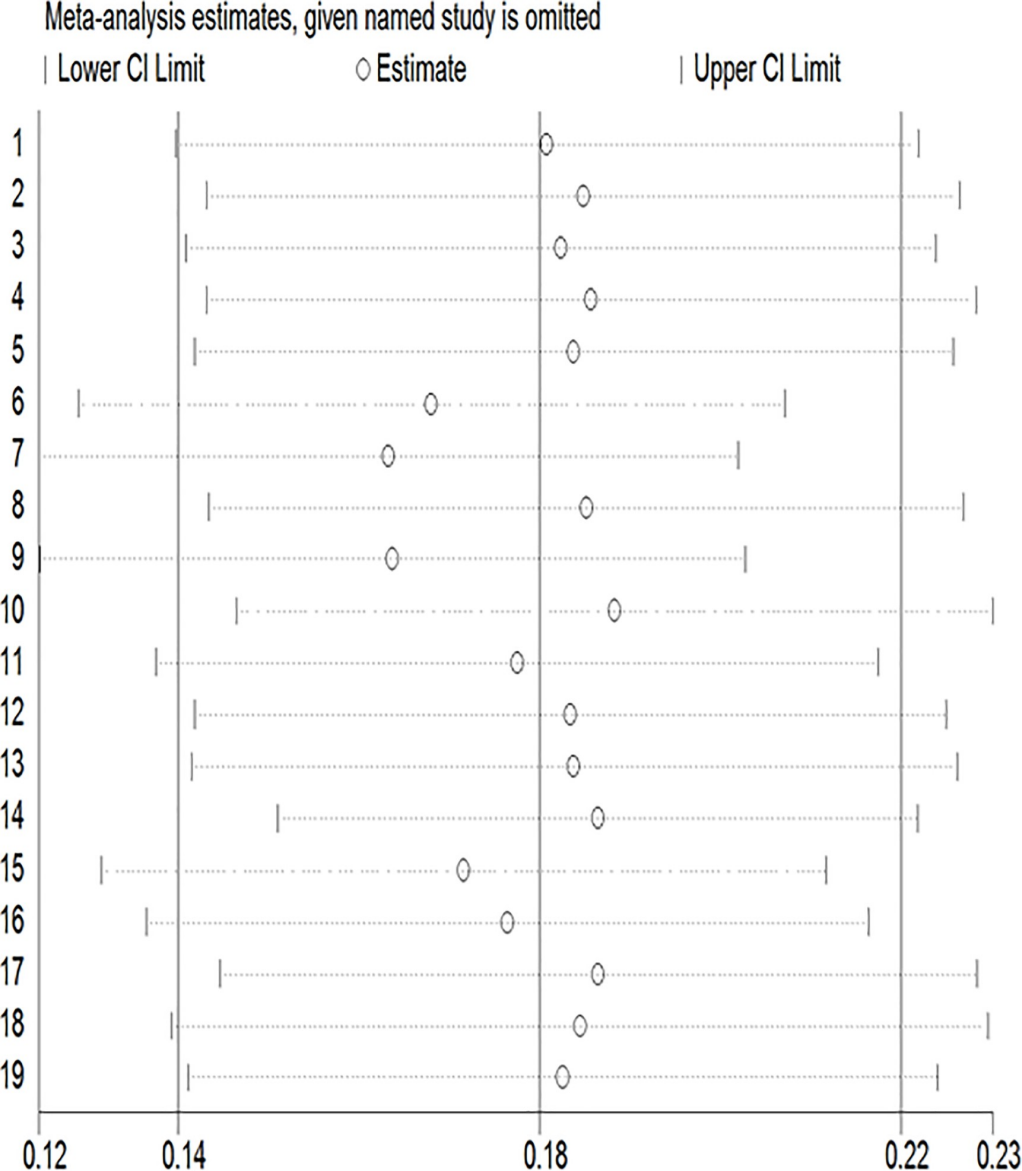
Sensitivity analysis between postnatal depressions studies included in a meta-analysis.

Accordingly, a pooled prevalence from sub-group analysis showed non-sub-Saharan countries had the highest prevalence of PND (PND = 44.05%: 95% CI; 33.77, 54.33, 2 studies from Egypt). Low-income African countries had higher PND (PND = 19.94%; 95%CI; 15.36, 24.52) prevalence compared with middle-income countries. Depression prevalence was found to be higher in the 1^st^ 96 weeks (PND = 41.46%; 95% CI: 25.78, 57.14) (Table 2). Sensitivity analysis showed that none of the studies substantially influenced the pooled estimates (Fig 5).

**Table 2:** Sub-analysis of postnatal depression in Africa (N = 19).

Variable for sub-analysis	Number of studies	Sample size (N)	Pooled prevalence (95%CI) random effect model
**Geography**			
Sub-Saharan countries	14	18,030	13.49(11.35,15.63)
Non-sub-Saharan countries	2	490	44.05(33.77,54.33)
Ethiopia	3	4,150	15.78(9.36,22.21)
**The income of the countries**			
Low-income	10	4,149	19.94(15.36, 24.52)
Middle-income	9	19,797	12.35 (10.13, 14.57)
**Time of depression measurement**			
Measured in the first 12 weeks	9	19,782	11.82(10.08, 13.55)
Measured in the first 48 weeks	8	3,764	17.22(14.33, 20.11)
Measured in the first 96 weeks	2	400	41.46(25.78, 57.14)
**Study setting**			
Community based	9	18,817	17.30 (14.45, 20.16)
Health institution based	10	5129	15.71(11.59, 19.82)
**Study design**			
Follow-up study	7	20,216	9.00 (7.12, 10.87)
Cross-sectional study	12	3,730	23.11 (17.75,28.47)
**Sample size**			
< = 384	7	1,684	28.03(15.49, 40.56)
>384	12	22,262	12.49(10.21, 14.79)
**Year of Publication**			
2008–2010	3	1,814	13.52(9.77, 17.27)
2011–2013	2	1,308	10.29(7.45, 13.13)
2014–2016	7	2,176	26.07(14.97, 37.16)
2017–2018	7	18,648	14.55(9.03, 20.08)
**Type of screening tool used**			
EPDS	6	19,277	16.41(8.89, 23.94)
PHQ-9	7	5,490	22.83(15.76, 29.89)
DSM-IV	2	1,291	11.31(9.25, 13.38)
Other(CES-D, SRQ-20, PDQ)	4	1,535	15.25(5.96, 24.54)

### Factors affecting postnatal depression

In the random effect model, the following factors were identified as a risk factor for PND. The odds of PND was higher among women with a history of the poor obstetric condition (such as Hyperemesis Gravidurum and cesarean section delivery) (Pooled odds Ratio (POR) = 1.72;95% CI: 1.36, 2.17; *I*^*2*^ *= 70*.*7%*). A history of adverse birth and infant health outcomes (such as low birth weight, preterm, stillbirth or infant loss after delivery) (POR = 2.38; 95% CI: 1.56, 3.64; *I*^*2*^ *= 74*.*5%*) were associated with an increased odds of PND. A history of maternal health problems during pregnancy (such as hypertension, gestational diabetes, HIV/AIDS, TB, and other health problems during pregnancy) (POR = 2.75; 95% CI; 1.89, 3.98; *I*^*2*^ *= 81*.*7%*) was associated with the risk of PND. Women with inadequate support from husband, family or any other source during pregnancy (POR = 2.06; 95% CI; 1.05, 4.05; *I*^*2*^ *= 83*.*2%*) were more likely to had PND compared to those who had good support.

**Fig 6:**
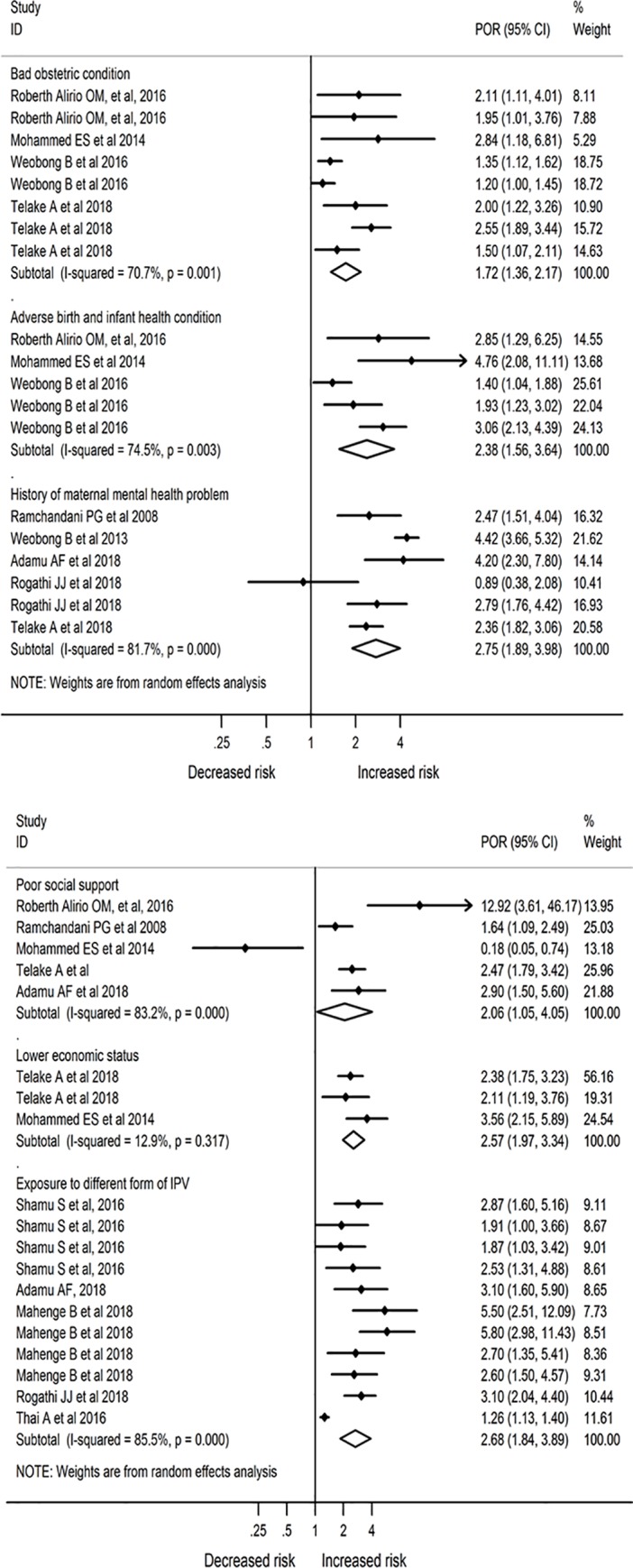
Factors affecting the occurrence of postnatal depression in Africa (N = 19; random effect).

The result also showed that low economic status (explained by financial hardship or low perceived wealth, experienced hunger in the past months) was significantly increased the odds of PND (POR = 2.57; 95% CI: 1.97, 3.34; *I*^*2*^ *= 12*.*9%*). Exposure to different forms of violence, such as physical, sexual, and psychological, was also significantly affected the occurrence of PND. Accordingly, the odds of depression among mothers who had exposure to a different form of IPV was 2.68 (POR = 2.68; 95% CI: 1.84, 3.89; *I*^*2*^ *= 85*.*5%*) times higher (Figs 5 & 6).

## Discussion

This systematic review and meta-analysis assessed the prevalence and factors affecting postnatal depression among postnatal women in Africa. In this review, a small number of studies with a high level of heterogeneity found that one in five postpartum mothers were likely to have PND in the continent. A history of the poor obstetric condition, adverse birth, and infant health outcomes, exposure to a different form of IPV, poor maternal health condition, and social support was found to increase the odds of PND.

This estimated prevalence was in line with a review conducted on perinatal depression in low and middle-income countries that showed nearly one-fifth (19%) of postnatal women had depression [26]. However, this review differs from the current in terms of geographical coverage, population, and year of publication. The highest pooled prevalence was reported from two studies in Egypt that might be due to a small sample size and their cross-sectional nature [78]. The Sub-Saharan countries had a high prevalence of PND likely due to economic struggle that leads to stress [79]. Postnatal depression prevalence appeared to increases the time of screening after delivery increased. It has been implicated that depression symptoms increased in the first two to 12 weeks because of hormonal fluctuation and the new environment of maternity [35, 80]. However, the interpretation for these estimates should account for the window of measurement as a more comprehensive window predicts more significant prevalence.

The pooled estimate of PND in Africa also differed by sample size included in the studies. Studies with larger sample size estimated lower prevalence as compared to studies with small sample sizes, and this is mostly true as precise estimation depends on the adequacy of sample size. The other variation in pooled estimate was accounted for the study type, cross-sectional and community-based studies relatively estimated bigger prevalence. Overestimation is a major limitation of cross-sectional studies. Similarly, as depression affects health-seeking behavior, women with depression tend to stay at home, and this might slightly increase prevalence estimates in community-based studies. The current estimate was higher compared with a review of longitudinal studies on perinatal depression that showed 13.1% of postnatal women had signs of depression [81]. In the previous review, longitudinal studies that followed women starting from pregnancy to postpartum period were included. During this course of follow-up, those who were depressed during pregnancy might have treated, and this might reduce the risk of recurrent depression in the postnatal period.

In this review, a history of poor obstetric and adverse birth and infant health conditions has significantly increased the odds of PND. This finding is supported by a systematic review and meta-analysis conducted in low-and middle-income countries [3]. The possible reason might be women with poor obstetric conditions are likely to feel guilty or ashamed [82], which leads to depression. Similarly, a history of adverse birth and infant conditions conceptualized as a stressful experience is mostly linked with depressive symptoms [1, 83, 84].History of maternal mental health problems significantly increased the odds of current PND. This finding was in line with a systematic review and meta-analysis of common perinatal mental disorders conducted in low and middle-income countries [6]. Women with a history of mental disorder are more likely to lose their positive affect [85], practice rumination and develop a negative cognitive style in their life that could also persist throughout the continuum of pregnancy [86, 87].

In the current review, poor social support was positively associated with the odds of PND. This finding was supported by a systematic review and meta-analysis carried out on the prevalence of postpartum depression and its effect in low and middle-income countries [57]. It has also been found that women who had weak support from close families at delivery or in the care of the newborn are less satisfied, stressful, and at a higher risk of depression [31].Being in lower economic status increased the risk of postnatal depression in this review. This finding was replicated in a systematic review and meta-analysis conducted in low and middle-income countries [3]. Women in low socio-economic status could become underprivileged due to scarcity of financial resources and insufficient health insurance, which leads to stress [27].

Exposure to different forms of IPV has increased the odds of postnatal depression in this review. This finding was supported by a systematic review and meta-analysis conducted in low and middle- income countries [88]. Exposure to IPV (physical, sexual, economical) could result in physical and social isolation; emotionally affect the abused women leading to low self-esteem, and disgusting lives [89, 90]that end up with depression. Depression during the postnatal period affects infant growth [14], breastfeeding practice [91] and under nutrition[22] through negatively affecting women’s interpersonal and parenting behavior [92]. The intimacy and interaction of the mother with her child would be affected, and the mother would fail to cope with her caring responsibility [93–95]. As a result, the child might suffer from diarrheal and other common childhood diseases [3]that might also lead to death. Depression also affects the mother’s quality of life [49].

So, depression screening during the postpartum period has significant importance. Findings from the current study, therefore, are helpful for clinicians, programmers, and policymakers to think of ways to integrate maternal mental health with routine maternal health services. The screening and treatment of depression should start by identifying an appropriate screening tool and setting the ideal time of testing. Early detection and treatment of PND would help to minimize its further re-occurrence and potential adverse health outcomes among the mothers and their children.

This review has the following limitations. Only English language reviews were included, and this might introduce publication bias. Moreover, the extent of heterogeneity among the included studies was high, which can be attributed to differences in methodology, study period, type of screening tool, and other unexplained variations. The other limitation of this review is that as only studies from 10 African countries were included, it might be difficult to generalize to the whole countries in Africa.

## Conclusion

This systematic review and meta-analysis found that PND could be arguably prevalent in the African continent based on a small number of published studies with inherently heterogeneous estimates. We also found that the prevalence of PND varied across different characteristics of the studies included in the review. Postnatal women with a history of the poor obstetric condition, adverse birth and infant health outcomes, mental health conditions, poor social support, and exposure to a different form of IPV were more likely to have PND. Based on this review, an early screening of postnatal depression and taking prompt intervention would save the mother and her baby from different forms of morbidity. This review also implicated that there is a shortage of robust studies in Africa to produce generalisablity evidence.

## Supporting information

S1 Checklist (DOC)

S1 Database (DOCX)

## Abbreviations

CES-D 20: Center for Epidemiologic Studies depression scale 20
DM: (Diabetic Mellitus): DSM-IV: Diagnostic and Statistical Manual of mental disorders version 4;EPDS: Edinburgh Postnatal Depression Scale
HIV: (Human Immune Deficiency): IPV: Intimate Partner Violence, LAMICS: (Low and Middle-Income Countries)
NOS: New Castle Ottawa Scale
OR: Odds Ratio
PDQ: Pitt Depression Questionnaire
PHQ-9: Patient Health Questioner-9;PND: (Postnatal Depression)
POR: Pooled Odds Ratio
PRISMA: (Preferred Reporting Items for Systematic Review and Meta-analysis), RR (Relative Risk), SRQ-20 (Self Reporting Questioner)
WHO: World Health Organization
