# Supplementary material for: Epidemiology of postnatal depression and its associated factors in Africa: A systematic review and meta-analysis
Source: PLoS One. 2020 Apr 28;15(4):e0231940. doi: 10.1371/journal.pone.0231940 (PMC7188237; doi:10.1371/journal.pone.0231940)
Supplement: S1 Database — (DOCX) [file pone.0231940.s002.docx]

**Search strategy for data bases**

| **#** | **Data base** | | **Search builder** |
| --- | --- | --- | --- |
|  | **Search strategy for postnatal depression** | | |
| 1 | [PsycINFO](http://ezproxy.flinders.edu.au/login?url=http://ovidsp.ovid.com/ovidweb.cgi?T=JS&MODE=ovid&D=psyh&PAGE=main&NEWS=Y) | (exp POSTPARTUM DEPRESSION/) or (Depress*.tw,id.) AND (postnat* or postnatal wom?n or postpartum wom?n).tw,id.) AND ((exp Risk Factors/) or (risk*.tw,id.)) AND ((cross sectional* or case control* or nested-case contorl).mp.) : all Sort by: PublicationDate Filters: Publication date from 2007/01/01 to 2018/06/30; Humans; English; Female; Field: Title/Abstract | |
| 2 | [Scopus](http://ezproxy.flinders.edu.au/login?url=https://www.scopus.com/scopus/home.url) | ((Postnatal mothers) OR (Postpartum mothers) OR (mothers after birth)) AND ((Depression during postnatal period) OR (postnatal depression ) OR (depression after birth ) OR (postpartum depressive symptom) OR (depressive mood following birth)) AND ((risk factors) OR correlates OR (associated factors) OR predictors)) AND ((cross sectional*) OR survey OR (case control*) OR (nested case control*) OR (prospective follow up) OR ( follow up) OR (retrospective follow up)) : all Sort by: PublicationDate Filters: Publication date from 2007/01/01 to 2018/06/30; Humans; English; Female;Field: Title/Abstract | |
| 3 | **Emcare** | (exp POSTPARTUM DEPRESSION/) or (Depress*.tw,id.) AND (postnat* or postnatal wom?n or postpartum wom?n).tw,id.) AND ((exp Risk Factors/) or (risk*.tw,id.)) AND ((cross sectional* or case control* or nested-case contorl).mp.) : all Sort by: PublicationDate Filters: Publication date from 2007/01/01 to 2018/06/30; Humans; English; Female;Field: Title/Abstract | |
| 4 | [MEDLINE](http://ezproxy.flinders.edu.au/login?url=http://ezproxy.flinders.edu.au/login?url=http://ovidsp.ovid.com/ovidweb.cgi?T=JS&PAGE=main&D=ppezv&MODE=ovid&NEWS=N) | (exp POSTPARTUM DEPRESSION/) or (Depress*.tw,id.) AND (postnat* or postnatal wom?n or postpartum wom?n).tw,id.) AND AND ((exp Psychosocial Factors/ or exp Risk Factors/) or (risk*.tw,id.)) AND ((prospective cohort* or retrospective cohort* follow up* or longitudinal* or cross sectional* or case control* or nested-case control).mp.) : all Sort by: PublicationDate Filters:Publication date from 2007/01/01 to 2017/06/30; Humans; English; Female;Field: Title/Abstract | |
